# Growth Rate and Outcomes in Locally Recurrent Extremity and Truncal Soft Tissue Sarcoma

**DOI:** 10.1001/jamanetworkopen.2024.31530

**Published:** 2024-09-04

**Authors:** George Z. Li, Kenneth Seier, Li-Xuan Qin, Murray Brennan, Carol D. Morris, Aimee M. Crago, Samuel Singer

**Affiliations:** 1 Department of Surgery, Memorial Sloan Kettering Cancer Center, New York, New York; 2 Department of Epidemiology and Biostatistics, Memorial Sloan Kettering Cancer Center, New York, New York

## Abstract

**Importance:**

Improved prognostic tools are needed for patients with locally recurrent extremity or truncal soft tissue sarcoma (STS).

**Objective:**

To examine the association between average local recurrence (LR) growth rate and outcomes following resection of locally recurrent extremity or truncal STS.

**Design, Setting, and Participants:**

This retrospective cohort study used a prospectively maintained database from a single high-volume tertiary sarcoma referral center in the US to identify patients 16 years of age or older who underwent repeat resection of a locally recurrent extremity or truncal STS between July 1, 1982, and December 31, 2021. Patients with atypical lipomatous tumors, desmoid tumors, dermatofibrosarcoma protuberans, angiosarcomas, and prior or synchronous distant recurrence were excluded. Data were analyzed from November 1, 2022, to June 17, 2024.

**Exposure:**

Average LR growth rate, defined as the sum of recurrent tumor maximal diameters divided by the disease-free interval after index operation.

**Main Outcomes and Measures:**

The primary outcomes were cumulative incidences of disease-specific death (DSD), with death from other causes as a competing risk, and second LR, with death from any cause as a competing risk.

**Results:**

The study cohort included 253 patients (median [IQR] age, 64 [51-73] years; 140 [55.3%] male). The 5-year cumulative incidence of DSD after repeat resection was 29%. Multivariable analysis indicated that LR growth rate (hazard ratio [HR], 1.12 [95% CI, 1.08-1.18]; *P* < .001), younger age (HR, 0.98 [95% CI, 0.97-0.99]; *P* = .002), R1 or R2 margins (HR, 1.71 [95% CI, 1.03-2.84]; *P* = .04), high LR grade (HR, 2.90 [95% CI, 1.17-7.20]; *P* = .02), and multifocality (HR, 2.92 [95% CI, 1.70-5.00]; *P* < .001) were independently associated with higher incidence of DSD. Using the minimum *P* value method, the optimal cutoff for growth rate was found to be 0.68 cm/mo. Patients with values above this cutoff had higher 5-year incidences of DSD following repeat resection (63% vs 19%; permutation test *P* < .001) and higher amputation rates (19% vs 7%; *P* = .008). Only R1 margins were independently associated with higher incidence of second LR (HR, 1.81 [95% CI, 1.19-2.78]; *P* = .006).

**Conclusions and Relevance:**

In this cohort study of patients undergoing resection of a locally recurrent extremity or truncal STS, LR growth rate was independently associated with DSD. These findings suggest that patients with growth rates higher than 0.68 cm/mo who undergo LR resection may have high disease-specific mortality and amputation rates and should be considered for perioperative systemic therapy.

## Key Points

**Question:**

What are the factors associated with disease-specific survival after resection of locally recurrent extremity or truncal soft tissue sarcoma?

**Findings:**

This cohort study of 253 patients who underwent resection of a locally recurrent extremity or truncal soft tissue sarcoma identified an independent association between higher average local recurrence growth rate (>0.68 cm/mo) and greater risk of disease-specific death.

**Meaning:**

These findings suggest that patients with average local recurrence growth rates higher than 0.68 cm/mo may be at high risk for disease-specific death and should strongly be considered for systemic therapy and enrollment in clinical trials.

## Teaser

This cohort study examines the cumulative incidences of disease-specific survival and local recurrence and their associated factors among patients who underwent resection of locally recurrent extremity or truncal soft tissue sarcoma.

## Introduction

Over half of adult soft tissue sarcomas arise from the extremities or trunk.^1^ Surgical resection is the mainstay of treatment for patients with localized disease, but up to 20% of patients develop a local recurrence (LR) by 10 years after surgery alone, with factors such as neoplasm size, grade, and histological examination findings being associated with LR risk.^2^ Even with the addition of perioperative radiation for patients at high risk for LR,^3,4,5^ LR rates have remained in the 6% to 10% range at experienced high-volume centers with modern radiotherapy techniques.^6,7,8^

For patients with extremity or truncal soft tissue sarcoma who experience LR, disease management can be challenging. Salvage surgery with or without radiation and reirradiation may be offered, but operations for LR are often more difficult due to the reoperative and often irradiated surgical field, which is reflected in a higher amputation rate for patients with recurrences in the upper or lower extremity.^9^ Furthermore, patients who experience a LR are also at higher risk for subsequent distant recurrence and disease-specific mortality,^10^ suggesting that LR is often reflective of aggressive underlying tumor biology. Nevertheless, a subset of patients experience prolonged disease-free intervals (DFIs) or cure after resection of a LR. As such, more granular prognostic information is needed to guide treatment decisions in the locally recurrent setting.

Members of our group previously found that for retroperitoneal liposarcomas, higher average LR growth rate, defined as the sum of maximal tumor diameters of the LRs divided by the DFI from the index operation, was associated with worse outcomes following LR resection.^11^ This metric was attractive as a prognostic tool because it was a single number that factored in 3 important tumor biology characteristics: DFI, size of the LR, and, to some extent, multifocality. Here, we sought to evaluate whether LR growth rate of an extremity or truncal soft tissue sarcoma would be similarly associated with worse outcomes.

## Methods

### Patient Cohort

This cohort study received institutional review board approval and and a waiver for obtaining informed consent from Memorial Sloan Kettering Cancer Center, New York, New York. This report follows the Strengthening the Reporting of Observational Studies in Epidemiology (STROBE) reporting guideline.

We reviewed all patients 16 years of age or older who underwent curative-intent R0 resection (macroscopically complete with negative microscopic margins) or R1 resection (macroscopically complete with positive microscopic margins) of a localized soft tissue sarcoma of the upper extremity, lower extremity, or superficial trunk at a single US institution between July 1, 1982, and December 31, 2021. We included patients who had LR, did not have a prior or synchronous distant recurrence, and underwent resection of the LR. We excluded patients with desmoid tumors, dermatofibrosarcoma protuberans, and well-differentiated liposarcomas or atypical lipomatous tumors, as these tumors have unique treatment paradigms and very low rates of distant metastases and disease-specific death (DSD). We also excluded patients with angiosarcomas because the size of these occultly infiltrative and often multifocal tumors is difficult to quantify.

### Clinical Characteristics and End Points

The primary variable of interest was average LR growth rate, which was defined as the sum of the maximal diameters of the recurrent tumors in centimeters divided by the DFI. If no preoperative therapy was administered, LR tumor diameters were determined from the pathology report. If preoperative therapy was administered, LR tumor diameters were determined from pretreatment cross-sectional imaging based on the longest dimension measured in any plane. DFI was defined as the time between the index operation and the date of LR in months. Secondary variables included age at the time of LR surgery, sex, histological examination findings, LR tumor grade, LR tumor depth, whether the LR was multifocal, LR resection margin status, whether perioperative radiotherapy was used to treat the LR, and whether perioperative chemotherapy was used to treat the LR. Given the limited number of events and the large number of histological subtypes, histological examination findings were grouped into 3 risk categories based on known prognostic information after resection of the primary tumor. Histological subtypes at high risk for DSD are not necessarily also at high risk for LR, and vice versa (eFigures 1 and 2 in Supplement 1); thus, we chose to stratify based on incidence of DSD into 3 groups. Group 1 (low risk, 7-year incidence of DSD <15%) included fibrosarcoma, inflammatory myofibroblastic tumor, and myxoid or round cell liposarcoma. Group 2 (average risk, 7-year incidence of DSD ≥15% and <30%) included myxofibrosarcoma, leiomyosarcoma, synovial sarcoma, dedifferentiated liposarcoma, extraskeletal chondrosarcoma, epithelioid sarcoma, and sarcoma not otherwise specified). Group 3 (high risk, 7-year incidence of DSD ≥30%) included undifferentiated pleomorphic sarcoma, malignant peripheral nerve sheath tumor, pleomorphic liposarcoma, Ewing sarcoma, and rhabdomyosarcoma.

The primary outcomes of interest were cumulative incidences of DSD and second LR. Outcome events and date of last follow-up were obtained from a prospectively maintained sarcoma database. Medical records were reviewed for all living patients up to October 2022 for additional outcome events. Other nonsarcoma causes of death were treated as competing risks in the analysis of DSD, and any cause of death was treated as a competing risk in the analysis of second LRs. Patients who underwent a macroscopically incomplete R2 resection for their first LR were excluded from the analysis of second LRs. We also performed exploratory subgroup analyses on the association between average LR growth rate and incidence of DSD within histological risk groups.

### Statistical Analysis

Categorical variables were summarized using counts and percentages and compared between groups using Fisher exact tests. Continuous variables were summarized using medians with IQRs and compared using Wilcoxon signed rank tests. DSD was defined from time of first LR resection to death due to disease or to last follow-up, with death from other causes treated as a competing event. Second LR was defined as time from first LR resection to a second LR or to the last follow-up, with death due to any cause without LR treated as a competing risk. Cumulative incidence functions were used to generate graphs; univariable and multivariable Fine and Gray models were used to analyze DSD and second LR. Factors significant at *P* = .10 in univariable analysis were entered into multivariable analysis, and backward selection was used to determine a final model. Due to its clinical significance, histological examination findings, and our variable of interest, growth rate, were forced into all multivariable models. Due to the high correlation with growth rate by definition, tumor size and DFI were not considered in the same multivariable model as growth rate. The minimum *P* value method was used to select the optimal cut point of growth rate for DSD^12^ Permutation tests were used to assess the optimal cut point for DSD and LR. We used SAS, version 9.4 (SAS institute Inc) and R, version 4.3.1 (R Project for Statistical Computing) for all analyses. All tests were 2-sided, and *P* < .05 was considered statistically significant.

## Results

Between 1982 and 2021, 3211 patients underwent curative-intent resection of a primary soft tissue sarcoma in the extremity or trunk (median [IQR] age, 55 (40-68) years; 1464 (46%) female, and 1747 (54%) male), of whom 253 patients experienced LR without prior or synchronous distant recurrence and underwent reresection of the LR (Table 1). The median (IQR) age for the LR cohort was 64 (51-73) years, 113 patients (45%) were female, and 140 patients (55%) were male. Median (IQR) follow-up for survivors in the entire cohort was 6.1 (2.2-11.2) years from the time of primary resection. There were 4 histological subtypes (solitary fibrous tumor, extraskeletal osteosarcoma, alveolar-soft part sarcoma, and liposarcoma not otherwise specified) that were not represented in the LR cohort, as all of these patients either did not have LR or experienced distant recurrence first or synchronously. Among patients with LR, 96% (243 of 253) had an upper or lower extremity tumor, and 4% (10 of 253) had a truncal tumor. Of 60 patients with low-grade primary tumors, 12 had high-grade recurrences (2 extraskeletal chondrosarcomas, 1 fibrosarcoma, 1 leiomyosarcoma, 1 myxoid or round cell liposarcoma, 1 sarcoma not otherwise specified, and 6 myxofibrosarcomas), and conversely, 6 of 193 patients with high-grade primary tumors had low-grade recurrences (1 extraskeletal chondrosarcoma, 5 myxofibrosarcomas). Of the remaining 48 patients with low-grade primary tumors, 45 had low-grade recurrences, and 3 had unknown recurrence grade. Of the remaining 187 patients with high-grade tumors, 183 had high-grade recurrences, and 4 had unknown recurrence grade. In the total cohort, 1327 of 3211 (41%) received perioperative radiotherapy, and 117 of 253 in the LR cohort (46%) received perioperative radiotherapy. Amputation rates were 5% (173 of 3211) for primary tumors and 10% (24 of 253) for LRs. For patients undergoing amputation for LR, 4 of 24 (17%) had preoperative chemotherapy, and none had preoperative radiotherapy prior to amputation for LR, although 13 of 24 (54%) had previously received radiotherapy for their primary tumor. Two patients in the LR cohort had amputations for recurrences at prior amputation stumps, and the remaining 22 patients had amputations due to extensive neurovascular or soft tissue involvement that precluded limb salvage.

**Table 1.** Patient Characteristics for the Entire Cohort at Index Operation and for the Study Cohort at LR Operation

Variable	Participants, No. (%)	
	Index operation (n = 3211)	LR operation (n = 253)
**Patient characteristics**		
Age at index or LR surgery, median (IQR), y	55 (40-68)	64 (51-73)
Sex		
Female	1464 (46)	113 (45)
Male	1747 (54)	140 (55)
**Histological examination finding**		
Group 1 (low risk)	564 (18)	33 (13)
Fibrosarcoma	155 (5)	9 (4)
Low-grade fibromyxoid sarcoma	45 (1)	0
Myxo-inflammatory fibroblastic sarcoma	32 (1)	4 (2)
Other or unknown	78 (2)	5 (2)
Inflammatory myofibroblastic tumor	21 (1)	2 (1)
Myxoid or round cell liposarcoma	388 (12)	22 (9)
Group 2 (average risk)	1533 (48)	134 (53)
Myxofibrosarcoma	521 (16)	70 (28)
Leiomyosarcoma	338 (10)	10 (4)
Synovial sarcoma	336 (10)	19 (8)
Dedifferentiated liposarcoma	80 (2)	11 (4)
Extraskeletal chondrosarcoma	79 (2)	11 (4)
Myxoid	73 (2)	11 (4)
Mesenchymal	5 (0)	0
Dedifferentiated	1 (<0.1)	0
Epithelioid sarcoma	53 (2)	3 (1)
Sarcoma NOS	126 (4)	10 (4)
Group 3 (high risk)	988 (301)	86 (34)
UPS	685 (21)	63 (25)
MPNST	99 (3)	9 (4)
Pleomorphic liposarcoma	109 (3)	5 (2)
Ewing sarcoma	52 (2)	6 (2)
Rhabdomyosarcoma	43 (1)	3 (1)
Not in LR cohort	126 (4)	NA
Solitary fibrous tumor	70 (2)	NA
Extraskeletal osteosarcoma	46 (1)	NA
Alveolar-soft part sarcoma	9 (0)	NA
Liposarcoma NOS	1 (<0.1)	NA
Site		
Upper extremity	853 (27)	91 (36)
Lower extremity	2209 (69)	152 (60)
Trunk	149 (5)	10 (4)
Tumor grade		
Low	791 (25)	51 (20)
High	2419 (75)	195 (77)
Missing	1 (<1)	7 (3)
Index procedure type		
Limb-sparing resection	3038 (95)	250 (98.8)
Amputation	173 (5)	3 (1)
LR Procedure Type		
Limb-sparing resection	NA	229 (90)
Amputation	NA	24 (10)
Resection margin		
R0	2808 (87)	166 (66)
R1	403 (13)	72 (28)
R2	0	7 (3)
Missing	0	8 (3)
Perioperative radiotherapy		
Yes	1327 (41)	117 (46)
No	1884 (59)	136 (54)
Perioperative chemotherapy		
Yes	623 (19)	19 (8)
No	2588 (81)	234 (92)
Maximal tumor diameter, median (IQR), cm	6.6 (3.8-11.0)	4.2 (2.2-7.0)
Multifocal LR		
Yes	NA	41 (16)
No	NA	197 (78)
Missing	NA	15 (6)
Disease-free interval, median (IQR), mo	NA	19 (8-38)
Average LR growth rate, median (IQR), cm/mo	NA	0.2 (0.1-0.6)

Abbreviations: LR, local recurrence; MPNST, malignant peripheral nerve sheath tumor; NA, not applicable; NOS, not otherwise specified; UPS, undifferentiated pleomorphic sarcoma.

After a median (IQR) follow-up of 5.3 (2.4-10.6) years for patients who underwent LR resection, 84 of 253 patients died of disease, with a 5-year cumulative incidence of DSD of 29%. The results of univariable analysis indicated that factors associated with higher incidence of DSD included age at the time of LR surgery (hazard ratio [HR], 0.99 [95% CI, 0.97-1.00]; *P* = .02), grade of LR (HR, 2.51 [95% CI, 1.29-4.88]; *P* = .007), R1 and R2 LR resection margins vs R0 LR resection margins (HR, 1.91 [95% CI, 1.24-2.96]; *P* = .003), LR size (HR, 1.06 [95% CI, 1.04-1.09]; *P* < .001), DFI (HR, 0.98 [95% CI, 0.97-0.99]; *P* = .004), multifocal LR (HR, 2.40 [95% CI, 1.47-3.91]; *P* < .001), and average LR growth rate (HR, 1.14 [95% CI, 1.09-1.19]; *P* < .001) (Table 2).

**Table 2.** Univariable and Multivariable Competing Risks Analysis of Disease-Specific Death

Variable	Univariable analysis		Multivariable analysis	
	HR (95% CI)	*P* value	HR (95% CI)	*P* value
Average LR growth rate			NA	
≤0.68 cm/mo	1 [Reference]			
>0.68 cm/mo	4.31 (2.71-6.86)	<.001		
Average LR growth rate (continuous variable)	1.14 (1.09-1.19)	<.001	1.12 (1.08-1.18)	<.001
LR size	1.06 (1.04-1.09)	<.001	NA	
Multifocal LR	2.40 (1.47-3.91)	<.001	2.92 (1.70-5.00)	<.001
Disease-free interval	0.98 (0.97-0.99)	.004	NA	
Histological subgroup				
Low risk	1 [Reference]		1 [Reference]	
Average risk	1.31 (0.66-2.63)	.44	1.03 (0.41-2.60)	>.95
High risk	1.60 (0.77-3.33)	.21	1.31 (0.48-3.56)	.60
LR resection margin				
R0	1 [Reference]		1 [Reference]	
R1 or R2	1.91 (1.24-2.96)	.003	1.71 (1.03-2.84)	.04
LR grade				
Low	1 [Reference]		1 [Reference]	
High	2.51 (1.29-4.88)	.007	2.90 (1.17-7.20)	.02
Age at LR resection	0.99 (0.97-1.00)	.02	0.98 (0.97-0.99)	.002
Sex				
Male	1.14 (0.74-1.76)	.54	NA	
Female	1 [Reference]			
Site				
Lower extremity	1 [Reference]			
Upper extremity or trunk	1.12 (0.73-1.72)	.61	NA	

Abbreviations: HR, hazard ratio; LR, local recurrence; NA, not applicable.

**Figure 1.**
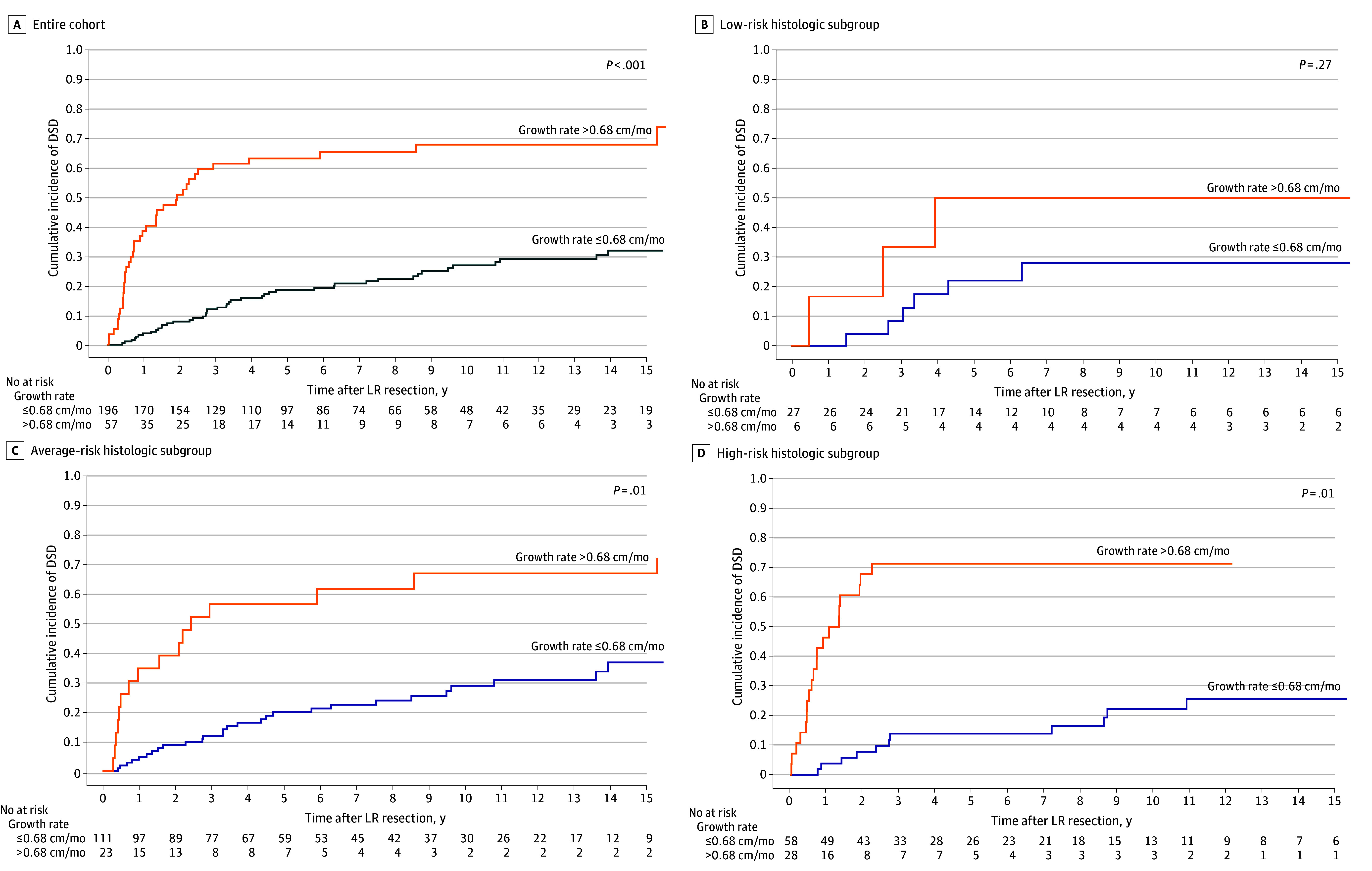
Cumulative Incidence of Disease-Specific Death (DSD) for Patients With Local Extremity Growth Rates Less Than or Equal to 0.68 cm/mo or Higher Than 0.68 cm/mo Survival time begins at time of local recurrence (LR) resection.

Using the minimum *P* value method, the optimal cut point for LR growth rate was determined to be 0.68 cm/mo, or 4 cm per 6 months (eFigure 3 in Supplement 1). Patients with a growth rate lower than or equal to 0.68 cm/mo had a 5-year incidence of DSD of 19%, while patients with a growth rate higher than 0.68 cm/mo had a 5-year incidence of DSD of 63% (HR, 4.31 [95% CI, 2.71-6.86]; *P* < .001) (Figure 1A). Patients with a growth rate higher than 0.68 cm/mo also had significantly higher amputation rates for resection of their LR (19% vs 7%; *P* = .008). In a multivariable analysis, factors independently associated with higher incidences of DSD included average LR growth rate as a continuous variable (HR, 1.12 [95% CI, 1.08-1.18]; *P* < .001), multifocality (HR, 2.92 [95% CI, 1.70-5.00]; *P* < .001), age at time of repeat resection (HR, 0.98 [95% CI, 0.97-0.99]; *P* = .002, LR grade (HR, 2.90 [95% CI, 1.17-7.20]; *P* = .02), and R1 or R2 margins vs R0 margins (HR, 1.71 [95% CI, 1.03-2.84]; *P* = .04) (Table 2). A multivariable model with LR size and DFI as separate variables instead of LR growth rate showed similar findings, with both LR size (HR, 1.06 [95% CI, 1.02-1.10]; *P* = .001) and DFI (HR, 0.98 [95% CI, 0.97-1.00]; *P* = .007) independently associated with higher incidence of DSD (eTable 1 in Supplement 1).

**Figure 2.**
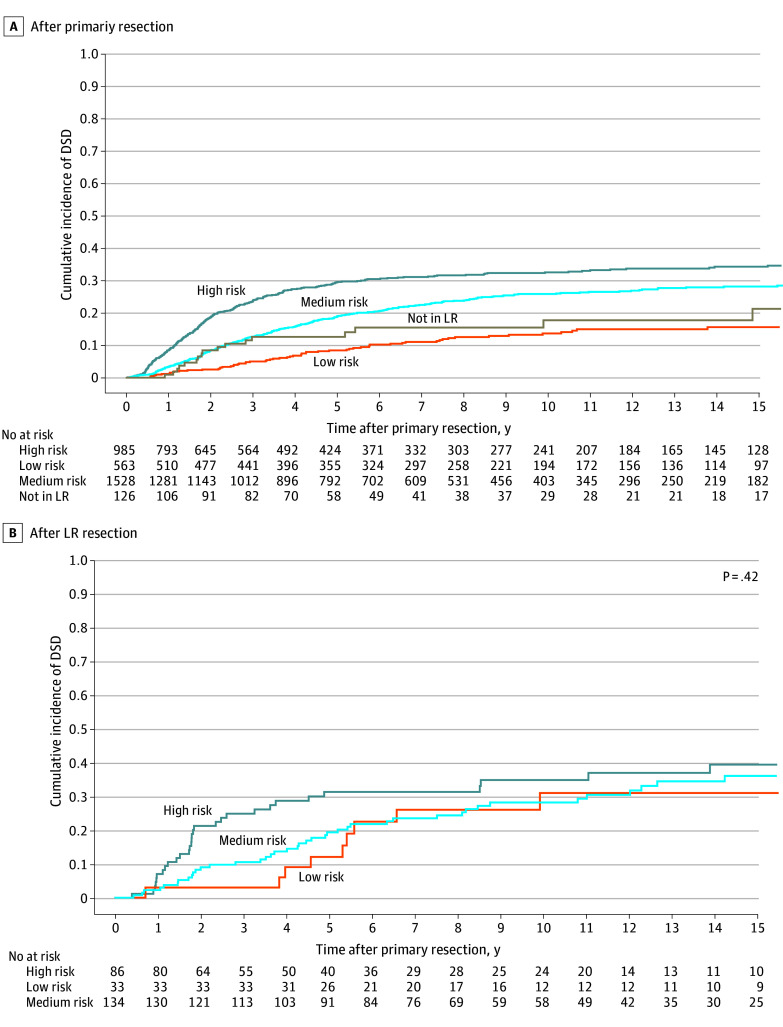
Cumulative Incidence of Disease-Specific Death (DSD) After Primary Tumor Resection (n = 3211) and Local Recurrence (LR) Resection (n = 253), Stratified by Histological Subgroup Survival time begins at time of index resection in panel A, and time of local recurrence resection in panel B.

Histological subgroups were defined by differences in DSD after primary resection (Figure 2A). However, for the LR cohort, histological subgroup was not associated with incidence of DSD after LR resection (Figure 2B). To further explore any potential interaction between histological examination findings and average LR growth rate, we performed exploratory subgroup analyses stratified by histological subgroup. These subgroups differed significantly in percentage of high-grade primary tumors (21% for low risk vs 77% for average risk vs 97% for high risk; *P* < .001), high-grade LRs (23% for low risk vs 80% for average risk vs 98% for high risk; *P* < .001), DFI (median [IQR], 25 [12-37] months for low risk vs 23 [11-43] months for average risk vs 12 [6-29] months for high risk; *P* = .006), and average LR growth rate (median [IQR], 0.2 [0.1-0.4] cm/mo for low risk vs 0.2 [0.1-0.5] cm/mo for average risk vs 0.3 [0.1-1.0] cm/mo for high risk; *P* = .005) (eTable 2 in Supplement 1). The differences in average LR growth rate were associated with differences in DFI, as LR size was not significantly different between histological subgroups (median [IQR], 4.5 [2.1- 6.0] cm for low risk vs 4.0 [2.0-7.0] cm for average risk vs 4.5 [2.5-8.0] cm for high risk; *P* = .46). Average LR growth rate greater than 0.68 cm/mo was significantly associated with higher incidence of DSD in the average-risk and high-risk subgroups but not in the low-risk subgroup (Figure 1B, C, and D).

For analysis of the second LR, we excluded 15 patients who had undergone gross incomplete R2 resections for their LR. Of the remaining 238 patients, 83 experienced a second LR, and the 5-year cumulative incidence of second LR was 36%. Univariable analysis indicated that the only factor associated with higher incidence of second LR was R1 vs R0 LR resection margins (HR, 1.76 [95% CI, 1.15-2.69]; *P* = .009) (Table 3). Local recurrence growth rate analyzed as a continuous variable was not associated with the incidence of second LR (HR, 0.98 [95% CI, 0.90-1.06]; *P* = .55). Multivariable analysis indicated that only R1 vs R0 LR resection margins was independently associated with higher incidence of second LR (HR, 1.81 [95% CI, 1.19-2.78]; *P* = .006).

**Table 3.** Univariable and Multivariable Competing Risks Analysis of LR

Variable	Univariable analysis (n = 238)		Multivariable analysis (n = 238)	
	HR (95% CI)	*P* value	HR (95% CI)	*P* value
Average LR growth rate	0.98 (0.90-1.06)	.55	0.97 (0.90-1.05)	.47
Multifocal LR	1.47 (0.89-2.45)	.14	NA	NA
Histological findings				
Low risk	1 [Reference]		1 [Reference]	
Average risk	0.64 (0.36-1.14)	.13	0.63 (0.36-1.09)	.10
High risk	0.73 (0.39-1.36)	.32	0.67 (0.37-1.24)	.20
LR resection margin				
R0	1 [Reference]		1 [Reference]	
R1	1.76 (1.15-2.69)	.009	1.81 (1.19-2.78)	.006
Age at LR resection	1.01 (1.00-1.03)	.07	NA	NA
Sex				
Male	1.03 (0.67-1.59)	.88	NA	NA
Female	1 [Reference]			
Site				
Lower extremity	1 [Reference]			
Upper extremity/trunk	1.44 (0.94-3.42)	.08	NA	NA
LR grade				
High	1.18 (0.69-2.03)	.55	NA	NA
Low	1 [Reference]			
Perioperative RT for LR				
Yes	1.05 (0.68-1.60)	.83	NA	NA
No	1 [Reference]			
Perioperative chemo for LR				
Yes	1.00 (0.41-2.44)	>.95	NA	NA
No	1 [Reference]			

Abbreviations: HR, hazard ratio; LR, local recurrence; NA, not applicable.

## Discussion

This cohort study found that more rapid average LR growth rate, younger age, LR grade, multifocality, and R1 or R2 LR resection were associated with higher incidences of DSD after re-resection in patients with locally recurrent extremity or truncal soft tissue sarcoma. LR growth rate, however, was not associated with incidence of a second LR, and only R0 vs R1 margin status was associated with differences in incidence of a second LR. Patients with LR growth rates less than or equal to 0.68 cm/mo (4 cm per 6 months) and low-grade recurrences showed low rates of DSD, and local control appeared to be the primary issue for these patients. Our results suggest that patients for whom an R0 margin can be achieved are good candidates for re-resection alone, whereas those for whom an R1 margin is predicted should be considered for adjunctive therapies, such as radiation, or for clinical trials of locoregional therapy, since the incidence of second LR in our study was high in this latter group of patients.

Patients with rapid LR growth rates (higher than 0.68 cm/mo) were at significant risk of DSD (>60% at 5 years). Such patients likely have aggressive tumors with a high risk of having microscopic metastatic disease that may be of greater concern than local control, which, in our study, was not associated with growth rate. Thus, in addition to surgical resection of the LR, patients with rapid LR growth rates should also be considered for systemic therapy, such as perioperative chemotherapy, clinical trials of immunotherapy with or without limb infusion,^13,14,15,16^ or clinical trials of targeted therapeutics.^17,18,19^ Patients should be strongly encouraged to participate in ongoing clinical trials, as current standard-of-care doxorubicin-based regimens unfortunately have low response rates of 17% to 30%.^20^ A preoperative approach may also be preferred, because the amputation rate for patients with average LR growth rates higher than 0.68 cm/mo also approached 20%, likely reflective of increased operative difficulty and anatomic constraints in the re-operative or recurrent setting.

A prior study from our institution, Memorial Sloan Kettering Cancer Center in New York, New York, identified short DFI, large LR tumor size, and histological grade to be associated with worse disease-specific survival in patients with LR of an extremity or soft tissue sarcoma, although neither the effect on second LR nor the impact of histological subtype was examined.^9^ Prior studies have also examined the prognostic utility of the treatment-naïve growth rate of primary soft tissue sarcomas,^21^ and ^18^F-fludeoxyglucose peak uptake^22^ and rate of uptake^23^ on positron emission tomography of primary and recurrent soft tissue sarcomas. Future work with larger multi-institutional datasets could investigate comprehensive models, including both LR growth rate and these other imaging variables.

Interestingly, we did not find that histological subtype was associated with DSD or second LR in the locally recurrent setting when stratified by DSD risk after primary tumor resection. One hypothesis is that the presence of LR selects for more aggressive subsets of low-risk histological subtypes. Furthermore, the exclusion of patients with prior or synchronous distant recurrences also removes the most aggressive subsets of high-risk histological subtypes. Thus, the clinical behavior in this specific patient cohort may be more uniform across histological subtypes, although histological grade remained independently associated with DSD in the locally recurrent setting. Within histological risk subgroups, an exploratory analysis identified that the average LR growth rate remained associated with incidence of DSD, except for the low-risk subgroup, which may not have had an adequate sample size to detect a small difference. Average LR growth rate was also highest in the high-risk subgroup, which appeared to be associated with differences in DFI across histological risk groups as opposed to tumor size.

### Limitations

Our study has several limitations. First, this was a retrospective single-institution study and is thus subject to selection bias and unmeasured confounders. Since the database we used dates back to 1982, some histological examination findings, such as fibrosarcoma have since been reclassified, and older cases do not have pathology slides readily available for re-review. Patients who experienced a LR but received treatment elsewhere may have been missed in our cohort. Some subgroups, such as patients with truncal tumors or patients who underwent R2 resection for LR, were too small in our cohort to assess for association with our primary outcomes on their own. Local recurrence growth rate also cannot be applied to tumors for which a size cannot be precisely obtained, such as angiosarcomas. Finally, we also need to validate our findings in an external dataset to confirm applicability to a broader patient population.

## Conclusions

The findings of this cohort study suggest that average LR growth rate along with LR histological grade can be used as a tool to select patients with LR who would potentially benefit from systemic therapy in addition to surgical treatment of their LR. In particular, high-grade recurrences with average growth rates higher than 0.68 cm/mo should be considered for systemic therapy prior to further procedures.

## Notes

Supplement 1. **eTable 1.** Multivariable Competing Risks Analysis of Disease-Specific Death With LR Size and Disease-Free Interval as Separate Variables **eTable 2.** Local Recurrence Cohort Characteristics Stratified by Histologic Subgroup **eFigure 1.** Cumulative Incidence of Disease-Specific Death After Primary Tumor Resection Stratified by Histologic Subgroup **eFigure 2.** Cumulative Incidence of Local Recurrence After Primary Tumor Resection Stratified by Histologic Subgroup **eFigure 3.** Minimum *P* Value Cutoff Analysis for Association Between Local Recurrence Growth Rate and Cumulative Incidence of Disease-Specific Death.

Supplement 2.
Data Sharing Statement

## References

[1] Brennan MF, Antonescu CR, Alektiar KM, Maki RM. Management of Soft Tissue Sarcoma. 2nd ed. Springer; 2016. doi:10.1007/978-3-319-41906-0

[2] Cahlon O, Brennan MF, Jia X, Qin LX, Singer S, Alektiar KM. A postoperative nomogram for local recurrence risk in extremity soft tissue sarcomas after limb-sparing surgery without adjuvant radiation. Ann Surg. 2012;255(2):343-347. doi:10.1097/SLA.0b013e3182367aa7 22143203 PMC5016830

[3] O’Sullivan B, Davis AM, Turcotte R, . Preoperative versus postoperative radiotherapy in soft-tissue sarcoma of the limbs: a randomised trial. Lancet. 2002;359(9325):2235-2241. doi:10.1016/S0140-6736(02)09292-9 12103287

[4] Pisters PW, Pollock RE, Lewis VO, . Long-term results of prospective trial of surgery alone with selective use of radiation for patients with T1 extremity and trunk soft tissue sarcomas. Ann Surg. 2007;246(4):675-681. doi:10.1097/SLA.0b013e318155a9ae 17893504

[5] Yang JC, Chang AE, Baker AR, . Randomized prospective study of the benefit of adjuvant radiation therapy in the treatment of soft tissue sarcomas of the extremity. J Clin Oncol. 1998;16(1):197-203. doi:10.1200/JCO.1998.16.1.197 9440743

[6] Danieli M, Barretta F, Fiore M, . Unplanned excision of extremity and trunk wall soft tissue sarcoma: to re-resect or not to re-resect? Ann Surg Oncol. 2021;28(8):4706-4717. doi:10.1245/s10434-020-09564-6 33511543

[7] Folkert MR, Singer S, Brennan MF, . Comparison of local recurrence with conventional and intensity-modulated radiation therapy for primary soft-tissue sarcomas of the extremity. J Clin Oncol. 2014;32(29):3236-3241. doi:10.1200/JCO.2013.53.9452 25185087 PMC4178522

[8] Alektiar KM, Brennan MF, Healey JH, Singer S. Impact of intensity-modulated radiation therapy on local control in primary soft-tissue sarcoma of the extremity. J Clin Oncol. 2008;26(20):3440-3444. doi:10.1200/JCO.2008.16.6249 18612160

[9] Eilber FC, Brennan MF, Riedel E, Alektiar KM, Antonescu CR, Singer S. Prognostic factors for survival in patients with locally recurrent extremity soft tissue sarcomas. Ann Surg Oncol. 2005;12(3):228-236. doi:10.1245/ASO.2005.03.045 15827815

[10] Lewis JJ, Leung D, Heslin M, Woodruff JM, Brennan MF. Association of local recurrence with subsequent survival in extremity soft tissue sarcoma. J Clin Oncol. 1997;15(2):646-652. doi:10.1200/JCO.1997.15.2.646 9053489

[11] Park JO, Qin LX, Prete FP, Antonescu C, Brennan MF, Singer S. Predicting outcome by growth rate of locally recurrent retroperitoneal liposarcoma: the one centimeter per month rule. Ann Surg. 2009;250(6):977-982. doi:10.1097/SLA.0b013e3181b2468b 19953716 PMC3248745

[12] Mazumdar M, Glassman JR. Categorizing a prognostic variable: review of methods, code for easy implementation and applications to decision-making about cancer treatments. Stat Med. 2000;19(1):113-132. doi:10.1002/(SICI)1097-0258(20000115)19:1<113::AID-SIM245>3.0.CO;2-O 10623917

[13] Keung EZ, Lazar AJ, Torres KE, . Phase II study of neoadjuvant checkpoint blockade in patients with surgically resectable undifferentiated pleomorphic sarcoma and dedifferentiated liposarcoma. BMC Cancer. 2018;18(1):913. doi:10.1186/s12885-018-4829-0 30249211 PMC6154892

[14] Bartlett EK, D’Angelo SP, Kelly CM, . Case report: response to regional melphalan *via* limb infusion and systemic PD1 blockade in recurrent myxofibrosarcoma: a report of 2 cases. Front Oncol. 2021;11:725484. doi:10.3389/fonc.2021.725484 34722269 PMC8554327

[15] Phase II study of neoadjuvant checkpoint blockade in patients with surgically resectable undifferentiated pleomorphic sarcoma and dedifferentiated liposarcoma. ClinicalTrials.gov identifier: NCT03307616. Updated April 5, 2024. Accessed July 18, 2024. https://clinicaltrials.gov/study/NCT03307616?term=NCT03307616&rank=1 10.1186/s12885-018-4829-0 PMC6154892 30249211

[16] A phase II study of concurrent systemic pembrolizumab and isolated limb infusion (ILI) with melphalan and dactinomycin for patients with locally advanced or metastatic extremity sarcoma. ClinicalTrials.gov identifier: NCT04332874. Updated July 11, 2024. Accessed July 18, 2024. https://clinicaltrials.gov/study/NCT04332874?term=NCT04332874&rank=1

[17] Pollack SM, Ingham M, Spraker MB, Schwartz GK. Emerging targeted and immune-based therapies in sarcoma. J Clin Oncol. 2018;36(2):125-135. doi:10.1200/JCO.2017.75.1610 29220291

[18] Phase 2 study of abemaciclib (LY2835219) in dedifferentiated liposarcoma. ClinicalTrials.gov identifier: NCT02846987. Updated December 28, 2023. Accessed July 18, 2024. https://clinicaltrials.gov/study/NCT02846987?term=NCT02846987&rank=1

[19] Phase I study evaluating combination therapy with the receptor tyrosine kinase inhibitor plx3397 and sirolimus in patients with unresectable sarcoma and phase II study in malignant peripheral nerve sheath tumors. ClinicalTrials.gov identifier: NCT02584647. Updated March 13, 2024. Accessed July 18, 2024. https://clinicaltrials.gov/study/NCT02584647?term=NCT02584647&rank=1

[20] Antman K, Crowley J, Balcerzak SP, . An intergroup phase III randomized study of doxorubicin and dacarbazine with or without ifosfamide and mesna in advanced soft tissue and bone sarcomas. J Clin Oncol. 1993;11(7):1276-1285. doi:10.1200/JCO.1993.11.7.1276 8315425

[21] Crombé A, Fadli D, Spinnato P, . Natural speed of growth of untreated soft-tissue sarcomas: a dimension-based imaging analysis. Eur J Radiol. 2022;146:110082. doi:10.1016/j.ejrad.2021.110082 34871937

[22] Fendler WP, Chalkidis RP, Ilhan H, . Evaluation of several FDG PET parameters for prediction of soft tissue tumour grade at primary diagnosis and recurrence. Eur Radiol. 2015;25(8):2214-2221. doi:10.1007/s00330-015-3654-y 25693667

[23] Dimitrakopoulou-Strauss A, Strauss LG, Schwarzbach M, . Dynamic PET 18F-FDG studies in patients with primary and recurrent soft-tissue sarcomas: impact on diagnosis and correlation with grading. J Nucl Med. 2001;42(5):713-720. 11337565

