# Supplementary material for: Growth Rate and Outcomes in Locally Recurrent Extremity and Truncal Soft Tissue Sarcoma
Source: JAMA Netw Open. 2024 Sep 4;7(9):e2431530. doi: 10.1001/jamanetworkopen.2024.31530 (PMC11375480; doi:10.1001/jamanetworkopen.2024.31530)
Supplement: Supplement 1. — eTable 1. Multivariable Competing Risks Analysis of Disease-Specific Death With LR Size and Disease-Free Interval as Separate Variables eTable 2. Local Recurrence Cohort Characteristics Stratified by Histologic Subgroup eFigure 1. Cumulative Incidence of Disease-Specific Death After Primary Tumor Resection Stratified by Histologic Subgroup eFigure 2. Cumulative Incidence of Local Recurrence After Primary Tumor Resection Stratified by Histologic Subgroup eFigure 3. Minimum P Value Cutoff Analysis for Association Between Local Recurrence Growth Rate and Cumulative Incidence of Disease-Specific Death. [file jamanetwopen-e2431530-s001.pdf]

Supplementary Online Content

Li GZ, Seier K, Qin LX, et al. Growth rate and outcomes in locally recurrent extremity and truncal soft tissue sarcoma. *JAMA Netw Open*. 2024;7(9):e2431530. doi:10.1001/jamanetworkopen.2024.31530

eTable 1. Multivariable Competing Risks Analysis of Disease-Specific Death With LR Size and Disease-Free Interval as Separate Variables

eTable 2. Local Recurrence Cohort Characteristics Stratified by Histologic Subgroup

eFigure 1. Cumulative Incidence of Disease-Specific Death After Primary Tumor Resection Stratified by Histologic Subgroup

eFigure 2. Cumulative Incidence of Local Recurrence After Primary Tumor Resection Stratified by Histologic Subgroup

eFigure 3. Minimum *P* Value Cutoff Analysis for Association Between Local Recurrence Growth Rate and Cumulative Incidence of Disease-Specific Death.

This supplementary material has been provided by the authors to give readers additional information about their work.

© 2024 Li GZ et al. *JAMA Network Open*.

eTable 1. Multivariable Competing Risks Analysis of Disease-Specific Death With LR Size and Disease-Free Interval as Separate Variables

	**Multivariable Analysis**	
Variable	HR (95% CI)	P value
LR size	**1.06 (1.02 – 1.10)**	**0.001**
Disease-free interval	**0.98 (0.97 – 1.00)**	**0.007**
Multifocality	**2.45 (1.39 – 4.33)**	**0.002**
Histology		
Low risk	Ref	-
Average risk	1.48 (0.54 – 4.02)	0.44
High risk	1.72 (0.59 – 5.02)	0.33
LR resection margin		
R0	Ref	-
R1/R2	**1.85 (1.10 – 3.12)**	**0.021**
LR grade		
Low	Ref	-
High	2.29 (0.94 – 5.61)	0.070
**Age at LR resection**	**0.98 (0.97 – 1.00)**	**0.007**

HR: hazard ratio; CI: confidence interval; LR: local recurrence

© 2024 Li GZ et al. *JAMA Network Open*.

eTable 2. Local Recurrence Cohort Characteristics Stratified by Histologic Subgroup

**Variable**	**Low-risk Histology n = 33 No. (%)**	**Average-risk Histology n = 134 No. (%)**	**High-risk Histology n = 86 No. (%)**	**P value**
Age at LR resection (years) Median (IQR)	59 (42 – 65)	65 (52 – 74)	65 (52 – 74)	0.12
Sex				0.70
Male	16 (48.5%)	76 (56.7%)	48 (55.8%)	
Female	17 (51.5%)	58 (43.3%)	38 (44.2%)	
Site				0.23
Upper extremity	8 (24.2%)	47 (35.1%)	36 (41.9%)	
Lower extremity	23 (69.7%)	83 (61.9%)	46 (53.5%)	
Trunk	2 (6.1%)	4 (3.0%)	4 (4.7%)	
**Primary tumor grade**				**<0.001**
**Low**	**26 (78.8%)**	**31 (23.1%)**	**3 (3.5%)**	
**High**	**7 (21.2%)**	**103 (76.9%)**	**83 (96.5%)**	
**LR tumor grade**				**<0.001**
**Low**	**23 (76.7%)**	**26 (19.8%)**	**2 (2.4%)**	
**High**	**7 (23.3%)**	**105 (80.2%)**	**83 (97.6%)**	
Missing	3	3	1	
LR margin				0.67
R0	21 (67.7%)	91 (70.0%)	54 (64.3%)	
R1/R2	10 (32.3%)	39 (30.0%)	30 (35.7%)	
Missing	2	4	2	
LR Size (cm) Median (IQR)	4.5 (2.1- 6.0)	4.0 (2.0 – 7.0)	4.5 (2.5 – 8.0)	0.46
**Disease-free interval (months), median (IQR)**	**25 (12 – 37)**	**23 (11 – 43)**	**12 (6 – 29)**	**0.006**
Multifocal LR				0.19
Yes	8 (26.7%)	23 (18.1%)	10 (12.3%)	
No	22 (73.3%)	104 (81.9%)	71 (87.7%)	
Missing	3	7	5	
**Average LR growth rate (cm/month), median (IQR)**	**0.2 (0.1 – 0.4)**	**0.2 (0.1 – 0.5)**	**0.3 (0.1 – 1.0)**	**0.005**

LR: local recurrence; IQR: interquartile range

© 2024 Li GZ et al. *JAMA Network Open*.

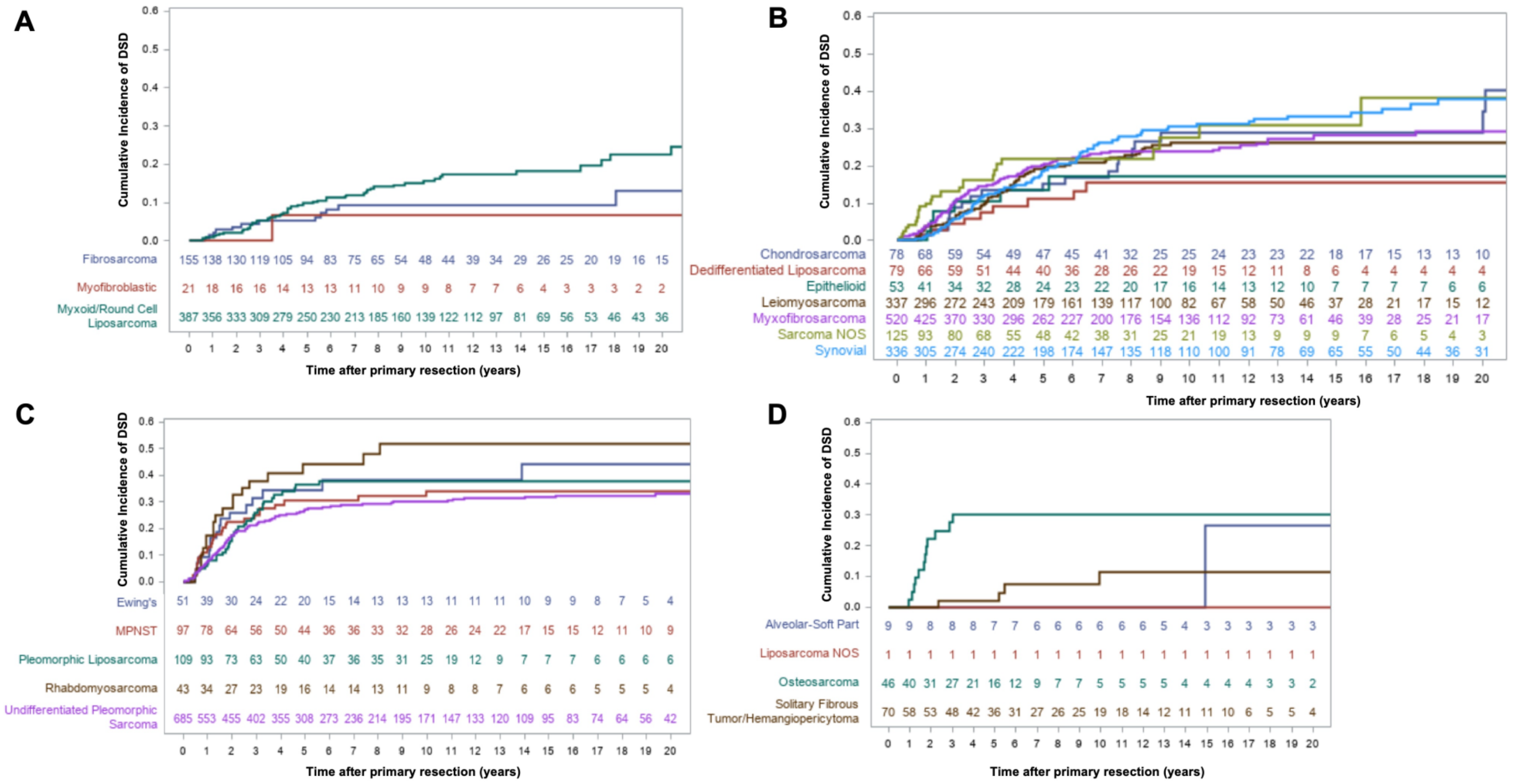

eFigure 1. Cumulative Incidence of Disease-Specific Death After Primary Tumor Resection Stratified By Histologic Subgroup: (A) low-risk subtypes, (B) average-risk subtypes, (C) high-risk subtypes, and (D) subtypes not represented in the locally recurrent cohort. Number of patients at risk for each group are displayed below the curves. Survival time begins at time of index resection.

© 2024 Li GZ et al. *JAMA Network Open*.

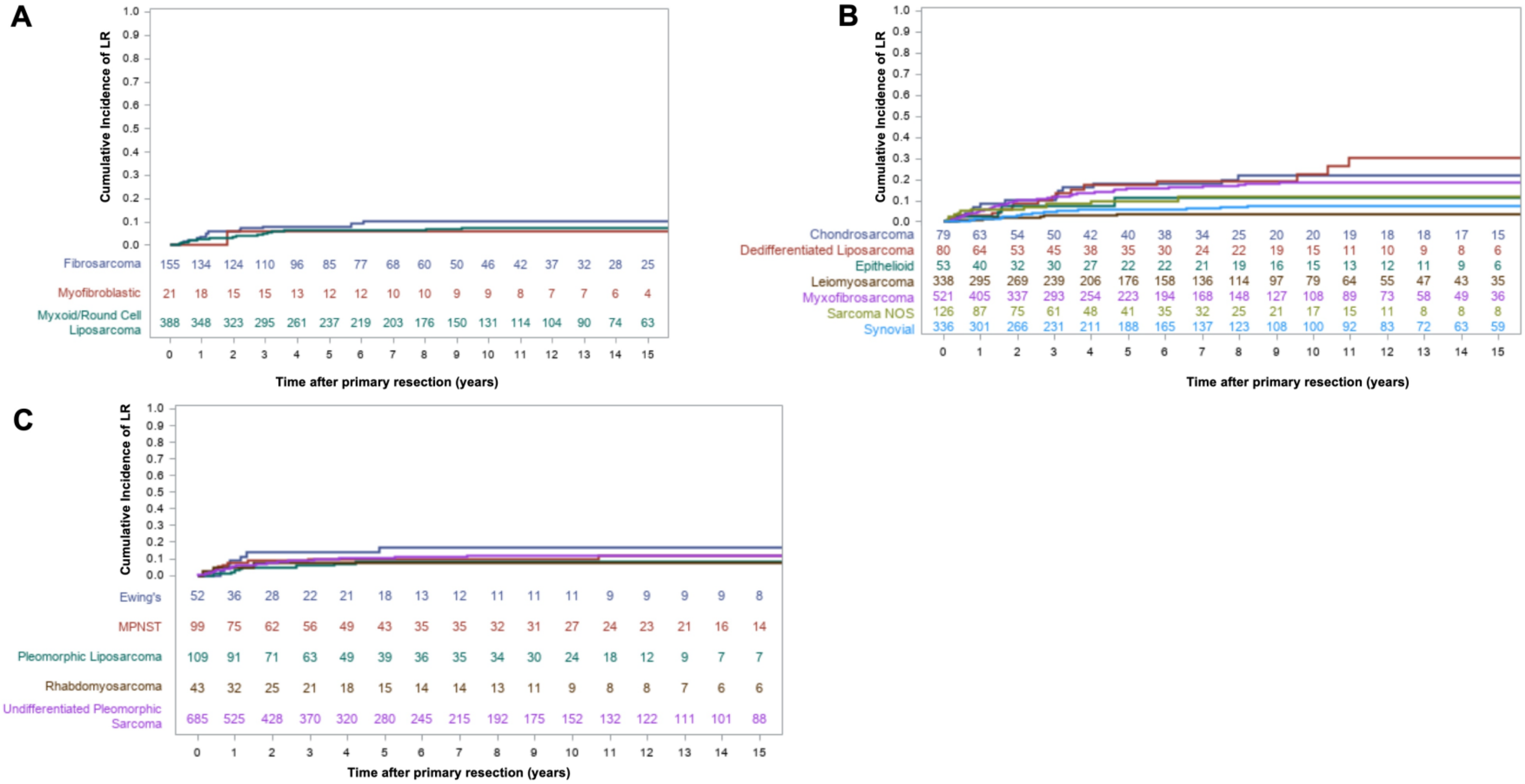

eFigure 2. Cumulative Incidence of Local Recurrence After Primary Tumor Resection Stratified by Histologic Subgroup: (A) low-risk subtypes, (B) average-risk subtypes, and (C) high-risk subtypes. Number of patients at risk for each group are displayed below the curves. Survival time begins at time of index resection.

© 2024 Li GZ et al. *JAMA Network Open*.

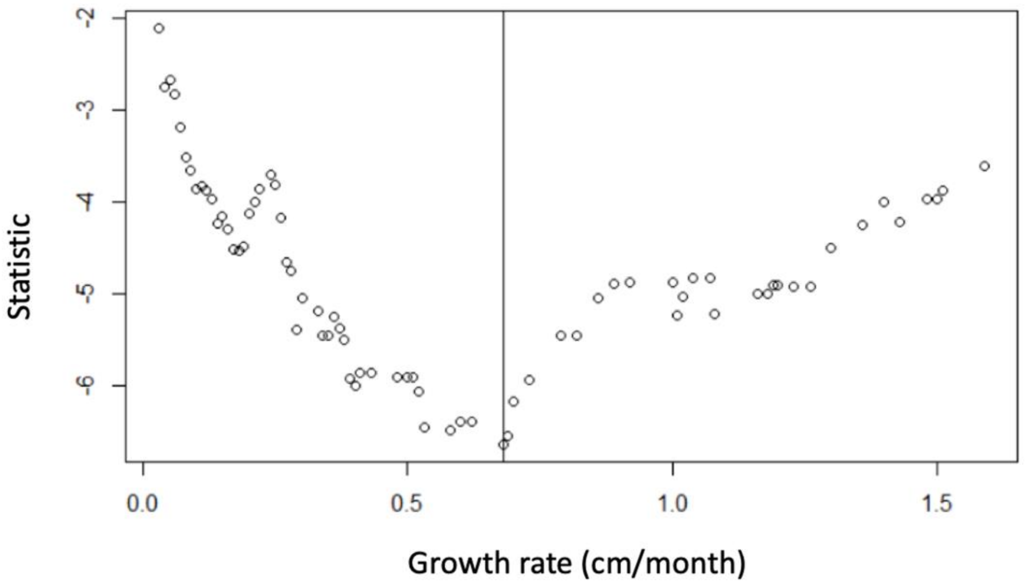

eFigure 3. Minimum *P* Value Cutoff Analysis for Association Between Local Recurrence Growth Rate and Cumulative Incidence of Disease-Specific Death. The vertical dashed line indicates the optimal cutoff.

© 2024 Li GZ et al. *JAMA Network Open*.
