# Supplementary material for: Growth Rate and Outcomes in Locally Recurrent Extremity and Truncal Soft Tissue Sarcoma
Source: JAMA Netw Open. 2024 Sep 4;7(9):e2431530. doi: 10.1001/jamanetworkopen.2024.31530 (PMC11375480; doi:10.1001/jamanetworkopen.2024.31530)
Supplement: Supplement 2. — Data Sharing Statement [file jamanetwopen-e2431530-s002.pdf]

Data Sharing Statement

Li. Growth Rate and Outcomes in Locally Recurrent Extremity and Truncal Soft Tissue Sarcoma. *JAMA Netw Open*. Published September 04, 2024.
doi:10.1001/jamanetworkopen.2024.31530

Data
Data available: Yes
Data types: Deidentified participant data
How to access data: singers@mskcc.org
When available: With publication

Supporting Documents
Document types: None

Additional Information
Who can access the data: researchers whose proposed use of the data has been approved
Types of analyses: for validation purposes
Mechanisms of data availability: after approval of proposal
